# Takotsubo Cardiomyopathy With Markedly Elevated Troponin Triggered by Acute Psychosis: A Case Report

**DOI:** 10.7759/cureus.98683

**Published:** 2025-12-08

**Authors:** Tarunjog Singh Kalra, Muhammad Shahid, Waseem Khamboo, Zoha Naeem, Hania Afsar

**Affiliations:** 1 Internal Medicine, Russells Hall Hospital, Dudley, GBR; 2 Cardiology, Russells Hall Hospital, Dudley, GBR

**Keywords:** apical ballooning syndrome, broken-heart syndrome, psychosis, stress induced cardiomyopathy, takotsubo cardiomyopathy (ttc)

## Abstract

Takotsubo cardiomyopathy (TTC) is an acute, reversible form of left ventricular dysfunction that mimics acute coronary syndrome (ACS), typically presenting with modest troponin elevation. We report a 38-year-old woman with a history of depression, who presented with acute psychosis requiring haloperidol sedation. Routine ECG monitoring revealed inferolateral ST-segment elevation, although she was asymptomatic at presentation and recalled only a non-specific burning chest discomfort three days prior. Laboratory tests demonstrated markedly elevated troponin I (>24,000 ng/L) and creatine kinase (5,931 U/L), unusual for TTC and suggestive of ACS. Bedside echocardiography revealed severe left ventricular systolic dysfunction with apical akinesia (ejection fraction (EF) ~35%), while coronary CT angiography showed unobstructed coronary arteries and a zero calcium score, confirming TTC. She was managed conservatively in the coronary care unit with bisoprolol and dapagliflozin, and psychiatric input guided safe antipsychotic use. Follow-up echocardiography demonstrated improvement in left ventricular function (EF 45%-49%), with resolution of symptoms.

This case highlights the rare occurrence of TTC with disproportionately high troponin levels and underscores the importance of multimodal imaging and multidisciplinary management in patients with psychiatric comorbidities to avoid misdiagnosis and unnecessary invasive procedures.

## Introduction

Takotsubo cardiomyopathy (TTC), also referred to as stress-induced cardiomyopathy, is characterized by transient systolic dysfunction of the left ventricle, most commonly involving apical ballooning. It is typically precipitated by intense emotional or physical stressors and presents clinically in a manner similar to acute coronary syndrome (ACS), with chest pain, ECG changes, and elevated cardiac biomarkers [1,2].

Troponin elevation in TTC is usually modest relative to the degree of left ventricular wall motion abnormality, and disproportionately high levels are uncommon [1,2]. Psychiatric illness, including acute psychosis, is a recognized precipitating factor, due to catecholamine surges and heightened sympathetic activation [1,3].

Understanding TTC is clinically important because it mimics ACS but usually does not involve obstructive coronary disease, and misdiagnosis may lead to unnecessary interventions. The pathophysiology involves catecholamine-mediated myocardial stunning, highlighting the link between acute stress, sympathetic activation, and transient cardiac dysfunction. We present a rare case of TTC associated with extremely elevated troponin levels, complicating differentiation from ACS.

## Case presentation

**Figure 1:**
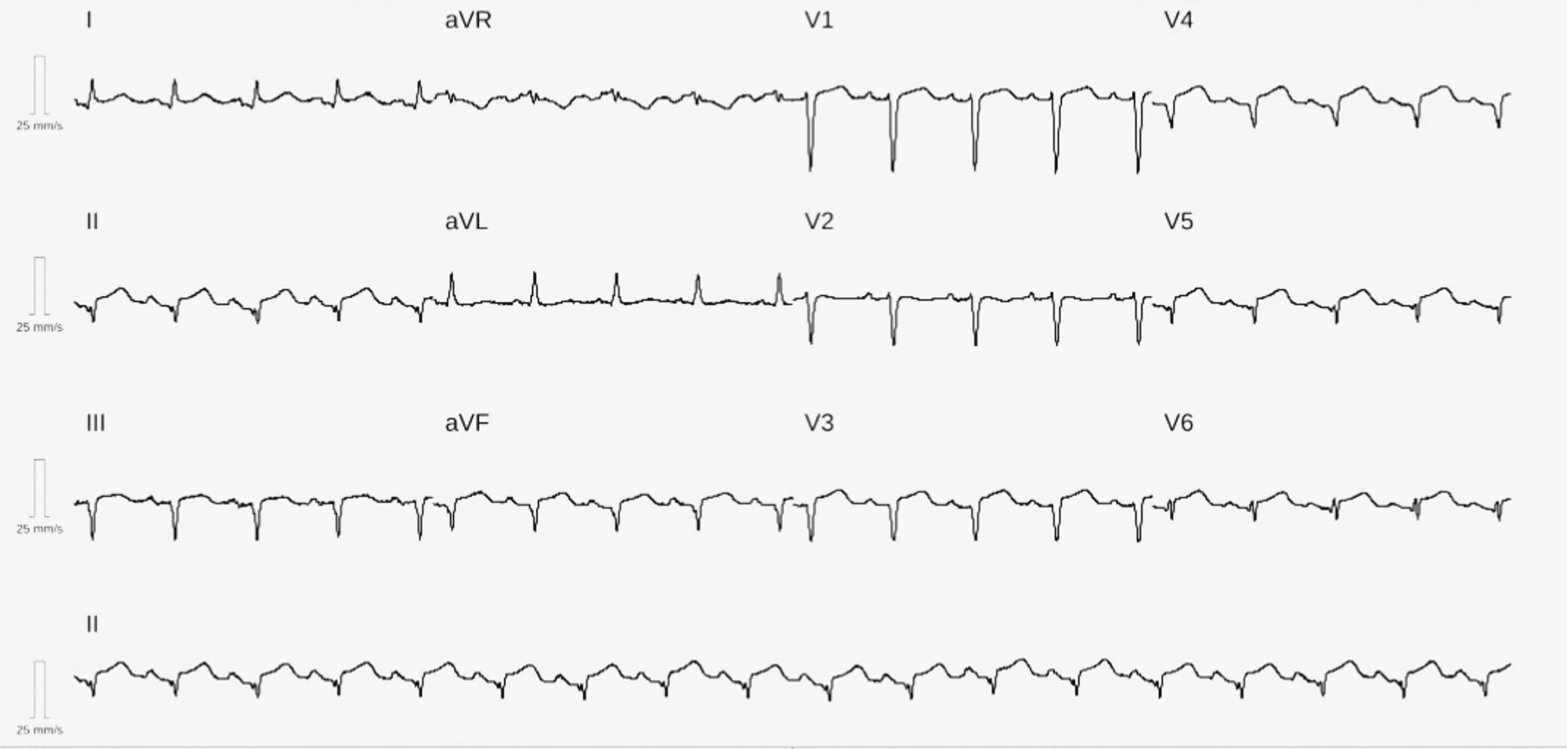
ECG shows inferolateral ST-segment elevation

A 38-year-old woman with a background of depression, maintained on sertraline 100 mg once daily, was admitted following an acute psychotic episode. On arrival, she exhibited severe agitation and aggression, requiring pharmacological sedation. She was administered haloperidol 5 mg intramuscularly, with good short-term behavioural control. Routine ECG revealed inferolateral ST-segment elevation (Figure 1). She remained asymptomatic at the time, but recalled experiencing a burning chest discomfort three days earlier. She denied cardiovascular risk factors, recreational drug use, or a family history of premature heart disease. On admission, her vital signs were stable: BP 108/76 mmHg, HR 102 bpm, temperature 36.9°C, and oxygen saturation 97% on room air. Physical examination was unremarkable.

During admission, she experienced a second psychotic episode, characterized by agitation, disorganised behaviour, and verbal aggression. This was managed with lorazepam 2 mg intramuscularly and de-escalation by the security team. Psychiatry later advised avoiding haloperidol due to QT-prolongation risk and recommended lorazepam 1-2 mg as needed, promethazine 25-50 mg as needed, and aripiprazole (initiated at 5 mg daily, with plans to titrate to 10 mg) for ongoing behavioural stabilization.

Laboratory investigations (Table 1) demonstrated markedly elevated cardiac biomarkers: troponin I 23,625 ng/L, peaking at 24,565 ng/L (delta 940 ng/L); creatine kinase 5,931 U/L; WCC 12.3 × 10⁹/L; and D-dimer 1,019 µg/L. The magnitude of troponin elevation initially raised concern for ACS. Bedside echocardiography showed severe left ventricular systolic dysfunction with apical akinesia and aneurysmal morphology, with an estimated ejection fraction (EF) of ~35% (Videos 1-3). Right ventricular function was preserved, and no apical thrombus was identified.

**Table 1:** Laborotory results MCV: Mean Corpuscular Volume; MCH: Mean Corpuscular Haemoglobin; MCHC: Mean Corpuscular Haemoglobin Concentration; eGFR: Estimated Glomerular Filtration Rate; CRP: C-Reactive Protein; HDL: High-Density Lipoprotein; LDL: Low-Density Lipoprotein; TSH: Thyroid Stimulating Hormone

Test	Patient Value	Reference Range
Sodium	136 mmol/L	135-145 mmol/L
Potassium	4.0 mmol/L	3.5-5.0 mmol/L
Urea	3.4 mmol/L	2.5-7.8 mmol/L
Creatinine	52 µmol/L	45-90 (F), 60-110 (M)
eGFR	>90 mL/min/1.73 m²	≥90 mL/min/1.73 m²
CRP	13 mg/L	<5 mg/L
Total Cholesterol	3.5 mmol/L	<5.0 mmol/L
Triglycerides	0.4 mmol/L	<1.7 mmol/L
HDL Cholesterol	1.2 mmol/L	>1.0 (M), >1.2 (F)
Non-HDL Cholesterol	2.3 mmol/L	<3.8 mmol/L
Calculated LDL	2.1 mmol/L	<3.0 mmol/L
Cholesterol:HDL Ratio	2.9	<4.0
TSH	1.40 mIU/L	0.4-4.0 mIU/L
Haemoglobin	116 g/L	130-170 (M), 120-150 (F)
White Cell Count	12.30 × 10⁹/L	4.0-11.0 × 10⁹/L
Platelets	227 × 10⁹/L	150-400 × 10⁹/L
Red Blood Cells	3.89 × 10¹²/L	3.8-4.8 (F), 4.2-5.4 (M)
Haematocrit	0.329	0.32-0.42 (F), 0.37-0.47 (M)
MCV	84.6 fL	80-100 fL
MCH	29.8 pg	27-33 pg
MCHC	353 g/L	320-360 g/L
Neutrophils	9.98 × 10⁹/L	2.0-7.5 × 10⁹/L
Lymphocytes	1.38 × 10⁹/L	1.0-3.5 × 10⁹/L
Monocytes	0.92 × 10⁹/L	0.2-0.8 × 10⁹/L
Troponin I (0 hours)	23,625 ng/L	<14 ng/L
Troponin I (3 hours)	24,565 ng/L	<14 ng/L
Troponin Delta (0-3 hours)	940 ng/L	<15 ng/L (acceptable delta)
Creatine Kinase	5,931 U/L	22-198 U/L
D-dimer	1,019 µg/L	<500 µg/L

**Video 1:** Two-chamber view of ECHO showing takutsubo cardiomyopathy on initial presentation

**Video 2:** Three-chamber view of ECHO showing takutsubo cardiomyopathy on initial presentation

**Video 3:** Four-chamber view of ECHO showing takutsubo cardiomyopathy on initial presentation

**Figure 2:**
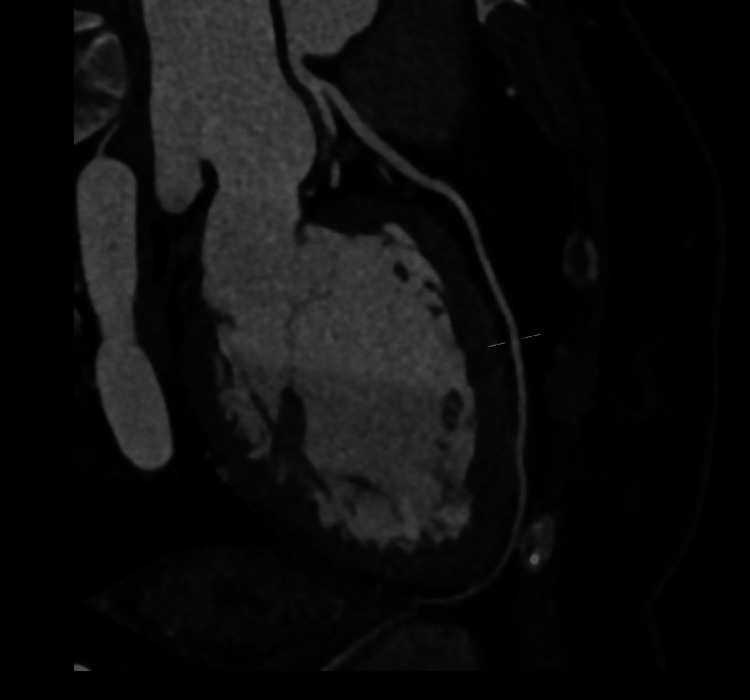
CT coronary angiogram showing patent left anterior descending artery

**Figure 3:**
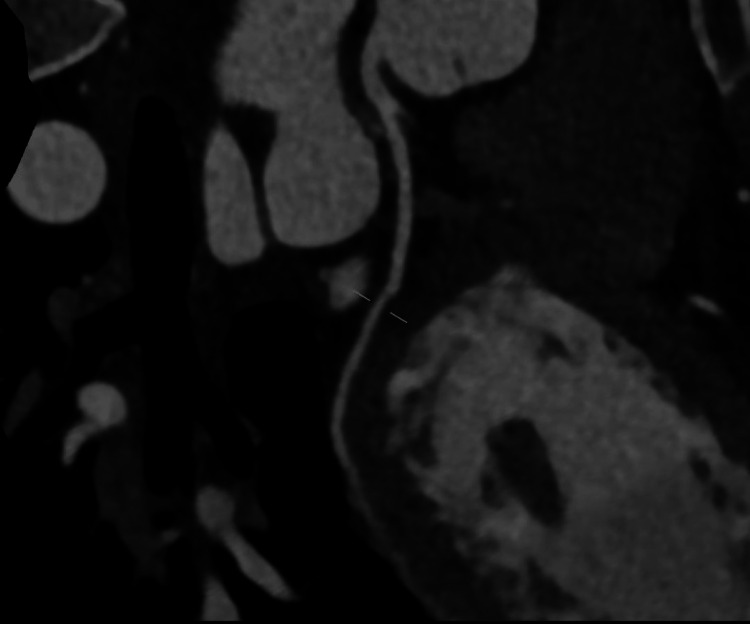
CT coronary angiogram showing patent left circumflex artery

**Figure 4:**
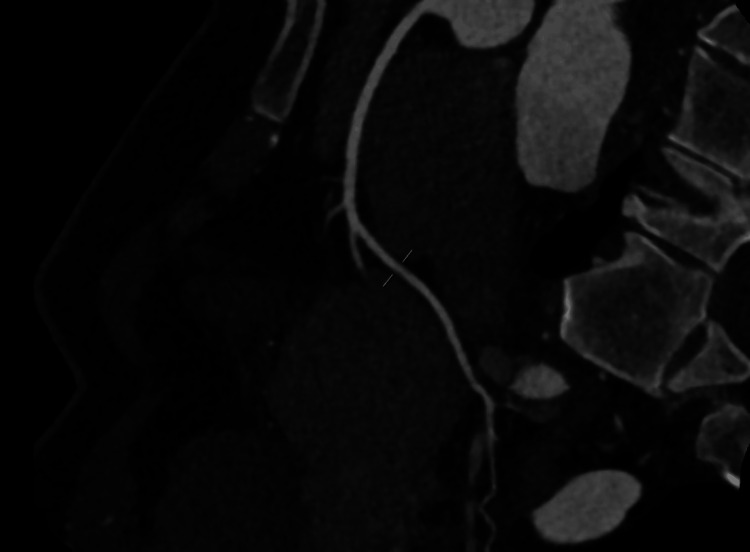
CT coronary angiogram showing patent right coronary artery

Coronary CT angiography demonstrated a calcium score of zero, right coronary dominance, and no evidence of coronary plaque or stenosis in the left anterior descending, left circumflex, or right coronary artery (Figures 2-4). CT head was unremarkable. She was admitted to the coronary care unit and commenced on bisoprolol 1.25 mg once daily, later uptitrated to 2.5 mg daily, and dapagliflozin 10 mg once daily, as part of guideline-directed therapy for left ventricular dysfunction. Continuous telemetry was used throughout her admission to monitor for arrhythmias, particularly given recent psychotropic use.

**Figure 5:**
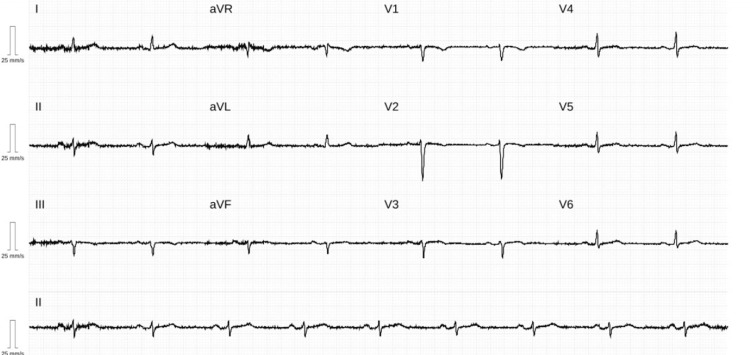
ECG on follow-up shows resolution of ST-elevation in inferior and lateral leads, with T-wave inversion in lead III, V1, V2, and V3

At early outpatient follow-up, repeat ECG (Figure 5) showed resolution of ST-elevation in inferior and lateral leads, with T-wave inversion in leads III, V1, V2, and V3. Echocardiography showed improvement in left ventricular systolic function (EF 45-49%) (Videos 4-6). She reported complete resolution of chest discomfort and demonstrated normal exercise tolerance. No further psychotic symptoms were reported, and she remained stable on aripiprazole 10 mg daily, with ongoing psychiatric oversight.

**Video 4:** Two-chamber view of ECHO showing improvement of ejection fraction on follow-up

**Video 5:** Three-chamber view of ECHO showing improvement of ejection fraction on follow-up

**Video 6:** Four-chamber view of ECHO showing improvement of ejection fraction on follow-up

## Discussion

TTC typically presents with transient left ventricular systolic dysfunction and ECG changes, mimicking ACS. Emotional and psychiatric stressors are well-recognized triggers [1]. Activation of the sympathetic nervous system in response to stress is thought to play a central role in the pathophysiology, leading to catecholamine-mediated myocardial stunning and the development of TTC.

Troponin elevation is common but usually modest relative to the extent of wall motion abnormality [2,3]. Our patient’s troponin exceeded 24,000 ng/L, which is exceptionally high for TTC and more typical of large myocardial infarction. This atypical biochemical profile highlights the diagnostic challenge and potential misclassification as ACS.

Multimodality imaging is critical in such cases. Echocardiography revealed classical apical akinesia, while CT coronary angiography excluded obstructive disease, confirming TTC. Management is primarily supportive, including heart failure therapy and avoidance of triggers. Prognosis is favourable, with recovery of systolic function within weeks in most patients.

Psychiatric comorbidity adds complexity, as agitation and psychotropic medications may exacerbate cardiac risk. Multidisciplinary collaboration between cardiology and psychiatry is therefore essential [1,3]. While the presentation strongly favours TTC, other differentials, such as myocarditis, type II MI, or drug-induced cardiomyopathy, were considered. Clinical features, imaging findings, and absence of systemic inflammation or causative drugs make these alternatives less likely.

## Conclusions

TTC can rarely present with markedly elevated troponin, mimicking ACS. Echocardiography and coronary imaging are essential to establish the diagnosis. Psychiatric illness may both trigger TTC and complicate management, necessitating multidisciplinary care.
